# Resolving the formidable barrier of oxygen transferring rate (OTR) in ultrahigh-titer bioconversion/biocatalysis by a sealed-oxygen supply biotechnology (SOS)

**DOI:** 10.1186/s13068-019-1642-1

**Published:** 2020-01-04

**Authors:** Xia Hua, Xin Zhou, GenLai Du, Yong Xu

**Affiliations:** 1 Jiangsu Co-Innovation Center of Efficient Processing and Utilization of Forest Resources, Nanjing Forestry University, Nanjing, 210037 People’s Republic of China; 2 College of Chemical Engineering, Nanjing Forestry University, No. 159 Longpan Road, Nanjing, 210037 People’s Republic of China; 3 Jiangsu Province Key Laboratory of Green Biomass-based Fuels and Chemicals, Nanjing, 210037 People’s Republic of China

**Keywords:** Sealed-oxygen supply technology (SOS), Ultrahigh-titer bioconversion, Oxygen transfer rate (OTR), Whole-cell catalysis, *Gluconobacter oxydans*

## Abstract

**Background:**

The critical issue in the competitiveness between bioengineering and chemical engineering is the products titer and the volume productivity. The most direct and effective approach usually employs high-density biocatalyst, while the weakened mass transfer and evoked foam problem accompany ultrahigh-density biocatalyst loading and substrate/product titer. In high-density obligate aerobic bioconversion, oxygen as electron acceptor is a speed-limiting step in bioprocesses, but sufficient oxygen supply will lead to the foaming which results in a significant reduction in oxygen utilization and the use of additional defoamers. In this study, we designed a novel sealed-oxygen supply (SOS) biotechnology to resolve the formidable barrier of oxygen transferring rate (OTR), for bio-based fuels and chemical production process.

**Results:**

Based on systemic analysis of whole-cell catalysis in *Gluconobacter oxydans,* a novel sealed-oxygen supply technology was smartly designed and experimentally performed for biocatalytic oxidation of alcohols, sugars and so on. By a simple operation skill of automatic online supply of oxygen in a sealed stirring tank bioreactor of SOS, OTR barrier and foaming problem was resolved with great ease. We finally obtained ultrahigh-titer products of xylonic acid (XA), 3-hydroxypropionic acid (3-HPA), and erythrulose at 588.4 g/L, 69.4 g/L, and 364.7 g/L, respectively. Moreover, the volume productivity of three chemical products was improved by 150–250% compared with normal biotechnology. This SOS technology provides a promising approach to promote bioengineering competitiveness and advantages over chemical engineering.

**Conclusion:**

SOS technology was demonstrated as an economic and universally applicable approach to bio-based fuels and chemicals production by whole-cell catalysis. The novel technology greatly promotes the competitiveness of bioengineering for chemical engineering, and provides a promising platform for the green and environmental use of biofuels.

## Background

It is well-known that biotechnology and bioengineering techniques possess several advantages over chemical methods, such as moderate reaction conditions, high chemoselectivity, and are environmentally friendly [1]. However, there exist still some major obstacles for the industrial application of biotechnology as compared with chemical engineering [2]. The critical issue in the competitiveness between bioengineering and chemical engineering is products titer and volume productivity [3]. The most direct and effective approach usually employs high-density biocatalyst [4], while the weakened mass transfer [5] and evoked foam problem inevitably accompany ultrahigh-density biocatalyst loading and substrate/product titer [6]. With the increasing demand for organic acids, whose global market share is only second to antibiotics and amino acids, the scale of whole-cell catalytic industry of aerobic microorganisms is increasing rapidly. However, to achieve the high-cell density and ultrahigh titer, whole-cell catalysis encounters several limiting factors such as oxygen transfer rate, foaming, and viscosity [7]. Thus, the industry of aerobic microorganisms is sending out ‘SOS’ to the society. The aerobic microorganisms applied in the industrial bioprocesses require large amounts of molecular oxygen as electron acceptor at the terminus of the cell respiratory electron transfer system [8], and re-oxidized nicotinamide adenine dinucleotide phosphate (NADPH) and/or flavin adenine dinucleotide (FADH) in electron transfer process to release abundant metabolic adenosine triphosphate (ATP) for cell maintenance, growth and catalytic reaction [9]. In bioprocesses, sufficient oxygen can effectively enhance catalytic efficiency and ensure the normal progress of reactions of aerobic microorganisms [10]. On the contrary, inadequate oxygen supply can severely inhibit regular cell growth and metabolism. Oxygen is an insoluble gas and thus, its solubility in water is low under normal conditions. Also, due to the complex and variable composition of culture in bioprocesses, the solubility of oxygen in broth is generally low than 0.21 mmol/L [11]. Moreover, the dissolved oxygen (DO) level is insufficient to support the effective transfer of oxygen between gas–liquid and solid–liquid phases in the broth [12]. Since there is minimal difference between saturated solubility and actual solubility of oxygen in the liquid phase, this implies shortage in the concentration difference of oxygen transfer rate (OTR). If oxygen is not added to the broth in time, the DO can maintain the normal physiological and biochemical functions of cells for only 15–20 s [13]. Therefore, ensuring an oxygen-enriched environment in the broth is a strict requirement for aerobic microorganisms [14].

Microbial respiration can utilize DO in the broth only to supply life activities or metabolic demands. Thus, strengthening OTR will be conducive to the improvement of DO level [15]. It is necessary to meet the high demand for oxygen by aerobic microorganisms through timely oxygen supply technology. Additionally, OTR is proportional to the oxygen transfer coefficient and the oxygen driving force [16]. The volumetric oxygen transfer coefficient of liquid phase represents the efficiency of oxygen transfer from gas phase to liquid phase, which means the difficulty of oxygen transfer. It determines the quality or adaptability of the basic design skills of the bioreactor involved in the regulation of microbial DO supply in the process of oxygen-consuming biological reaction [17]. The factors affecting oxygen transfer efficiency include stirring speed, air velocity, physical and chemical properties of the broth, foaming, structure design of the bioreactor, etc. For oxygen driving force, it represents the difference between saturated DO and actual DO in broth. The factors affecting oxygen driving force include temperature, properties of solutions, oxygen partial pressure, viscosity and so on [18]. Traditionally, the creation of an oxygen-enriched environment, which aims at enhancing OTR, mainly depends on the direct introduction of oxygen instead of air into the bioreactor [19]. However, unrestricted large-scale supply of pure oxygen not only leads to extremely low oxygen utilization, resulting in waste of resources and sharp increase in production costs, but also causes irreversible foam problem, which makes the timely updation of fresh gas difficult, and ultimately inhibiting microbial basal metabolism [20]. Moreover, several techniques have been employed in the bioreactor for strengthening OTR including elevated partial pressures of oxygen supplies [21], increased agitation rate [22], and modifying the vessel [23]. Additionally, it is feasible to add some oxygen carriers with high solubility to the broth. Irrespective of the operation, circumspect explorations are needed to prevent a severe condition from causing fatal damage to the relatively fragile microorganisms. Therefore, by supplementing the oxygen needed for aerobic respiration for microbial growth and metabolism, i.e., to maintain the balance between oxygen supply and consumption, we can effectively and economically improve the level of microbial transformation and fermentation.

In the whole-cell catalytic process of aerobic microorganisms, DO in the broth tends to be zero when OTR cannot meet the oxygen uptake rate (OUR) [24]. Consequently, DO cannot satisfy the respiratory intensity of the microorganisms, which results in the cells being in a suppressed and oppressed state. Hence, oxygen is the core element for aerobic microorganisms [25]. Based on these limitations and considering the cost economy and the rationality of industrial production, we improved the conventional bioreactor and designed a sealed oxygen supplied bioreactor (SOS-BR). For SOS-BR, a 99.9% pure oxygen cylinder, instead of air compressor, is connected to a stirred bioreactor to strengthen the oxygen driving force. Furthermore, a barometer is installed at the outlet and the tank pressure is controlled in the range of 0.03–0.05 MPa. The SOS-BR has several advantages including oxygen enrichment in the environment, high utilization rate of oxygen, and large partial pressure of oxygen. Bacterial strain *Gluconobacter oxydans* (*G. oxydans*) is a typical obligatory aerobic Gram-negative microorganism [26, 27]. Due to its strict regioselectivity and stereoselectivity incomplete oxidation, it has been widely employed in large-scale industrialized preparation of various biochemical products [28]. Additionally, *G. oxydans* processes a series of membrane-bound dehydrogenases, including four enzymes: ethanol dehydrogenase, aldehyde dehydrogenase, sorbitol dehydrogenase, and glucose dehydrogenase [27]. The function of these dehydrogenases is to bio-convert alcohol and aldehyde substrates to corresponding ketones or acids. *G. oxydans* has been used in the synthesis of glycolic acid [29, 30], 1,3-dihydroxyacetone [31], sorbose [32], and several sugar acids [33, 34]. Incomplete oxidizing strains, such as *G. oxydans* and *Gluconobacter frateurii* do not generate waste gas (CO_2_) in the physiological and biochemical processes. Hence, incomplete oxidized aerobic microorganisms are in line with the SOS-BR operation model and can be operated continuously for a long time. For other aerobic microorganisms, even if small levels of CO_2_ are produced during the bioprocess, the whole-cell catalysis in SOS-BR can be achieved as long as the gas pressure in the tank is controlled in a safe range by intermittent ventilation. In this paper, *G. oxydans* is the representative strain used to investigate the economic practicability and universal applicability of SOS-BR to aerobic microorganisms. The modified operation bioreactor provides a valuable reference to promote cost-effective production by oxygen consumption biocatalysis in factories.

## Results and discussion

### Design SOS system based on systemic analysis of whole-cell catalysis of *G. oxydans*

**Fig. 1:**
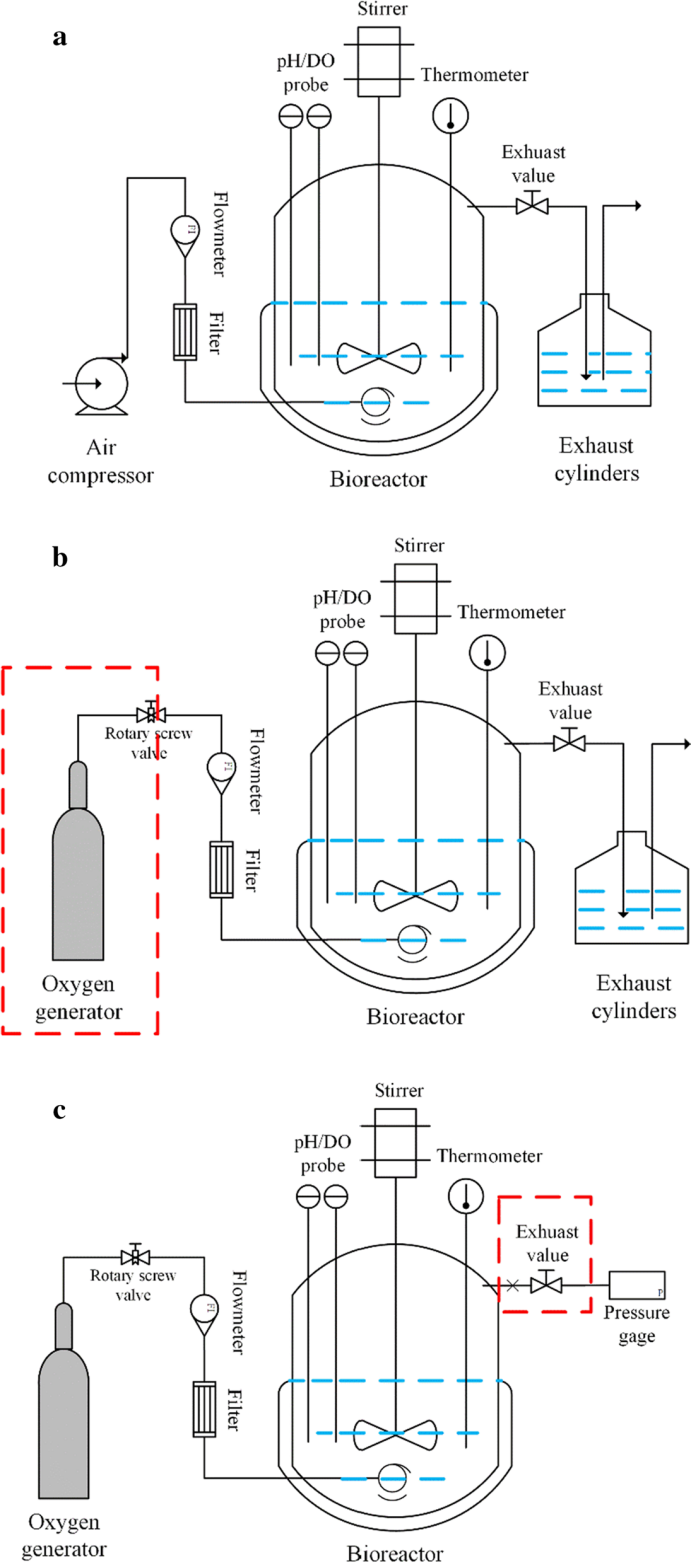
Three bioreactor operation models with different ventilation forms: **a** AS-BR, **b** OS-BR, **c** SOS-BR

Oxygen is the linchpin dynamics of oxidation reaction in the process of *G. oxydans* biocatalytic conversion to organic acid. Therefore, improving the mass transfer capacity of oxygen greatly contributes to catalytic oxidation. Moreover, in order to highlight the superiority of SOS-BR, we increased the initial cell inoculation quantity to 20 OD (190 g/L wet weight) which is the maximum conventional industry access quantity. Under these conditions, the whole-cell catalysis of *G. oxydans* was carried out in three bioreactors as shown in Fig. 1. However, in order to meet the requirements of 20 OD for industrial production, AS-BR could not satisfy the oxygen demand of cells. Apart from that, due to the high concentration of sugar in the reaction process, the viscosity of broth increased rapidly, thus making it difficult to eliminate the bubbles.

Conventionally, replacing air with pure oxygen is the quickest and simplest approach to maintain absolute oxygen-enriched conditions in a bioreactor. Although the DO level in this device was sufficient to sustain the aerobic demand of the cells, there were still two fatal obstacles. First, due to the continuous and uninterrupted oxygen supply, the utilization rate of oxygen was extremely low, which is not practical. Also, pure oxygen is expensive, a large amount of oxygen consumption will lead to a significant increase in production expenses, which is not economic. Second, the foaming issue is still inevitable, as the bubbles are hard to break and oxygen rises through the bubbles to the gas–liquid interface and released into the air. The most direct technique to solve this problem is to add an appropriate amount of defoaming agent. However, defoaming agents are not only expensive, but also affect the purity of the product, which is not suitable for large-scale industrial production.

Conversely, SOS-BR could conquer these defects. In SOS-BR, when the pressure in the tank reached the control interval, it indicated that there is enough oxygen in the bioreactor. At this time, the oxygen flow was compressed, and the oxygen utilization rate increased to over 90% while maintaining enough oxygen. When the microorganisms used oxygen quickly, the inlet ventilation flow was compensated accordingly. When the oxygen consumption rate was slow, the inlet ventilation flow was weakened correspondingly. This control model greatly debases the production cost and ensures full utilization of resources. Additionally, increasing the tank pressure will reduce the volume of bubbles, effectively restraining the formation of foam, and completely eliminating the bubble problem. Thus, SOS-BR is a flawless biotechnology technique to promote the industrialization of aerobic microorganisms.

### SOS performances in bio-oxidation of various oxy-compounds

*Gluconobacter oxydans* consists of four main enzymes: ethanol dehydrogenase, acetaldehyde dehydrogenase, glycerol dehydrogenase, and aldose dehydrogenase. Therefore, we chose 1,3-PG [35] and erythritol [36] as raw materials, which covered the main enzymes system of *G. oxydans* besides aldose dehydrogenase, and tested the practicability and universality of SOS-BR.

**Fig. 2:**
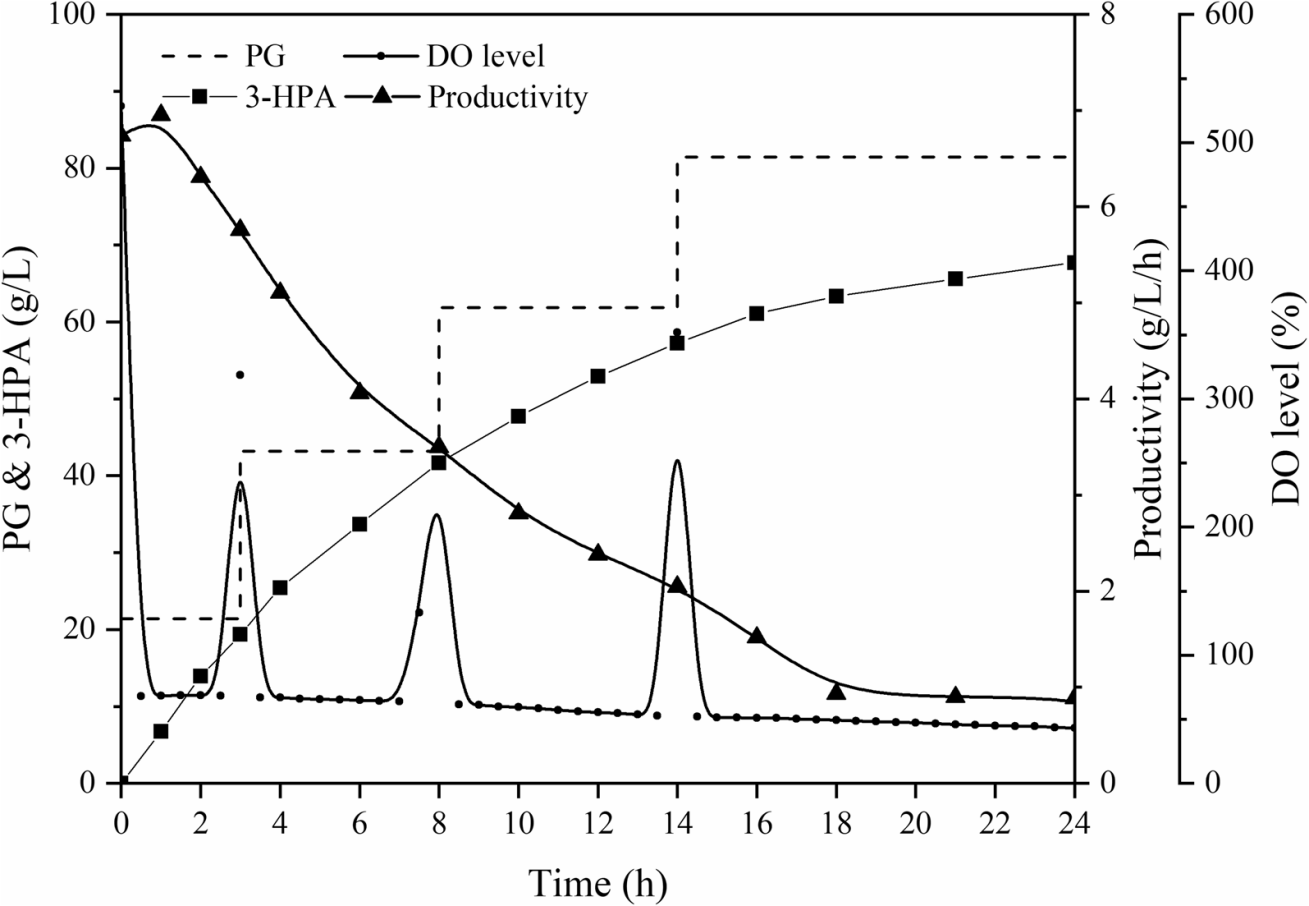
The whole-cell catalysis profile of 1,3-propylene glycol in SOS-BR

There are no reports on the preparation of 3-HPA by microorganism due to the toxicity of the substrates. The highest yield of 3-HPA was obtained by using genetic modification of *K. pneumoniae*. With *K. pneumoniae* DSM 2026 as catalyst, the maximum production of 3-HPA in one single batch was 83.8 g/L for 72 h, but the yield was only 1.2 g/L/h [37]. Moreover, using *K. pneumoniae* WM3, the maximum yield of 3-HPA was 2.0 g/L/h, but the production was only 48.9 g/L [38]. However, SOS-BR, a promising biotechnology technique can significantly increase both production and productivity of 3-HPA. As is shown in Fig. 2, in 24-h whole-cell catalysis, 67.7 g/L and 2.8 g/L/h 3-HPA was acquired, and the productivity exceeded the current highest level by about 40%. As for the one batch production, although 3-HPA production was less than 83.8 g/L, the fermentation time of the entire batch was 72 h, much longer than the 24 h of the catalysis using *G. oxydans*. In terms of productivity, there was significant difference between the two methods, and the results in SOS-BR were high by about 133.3%.

**Fig. 3:**
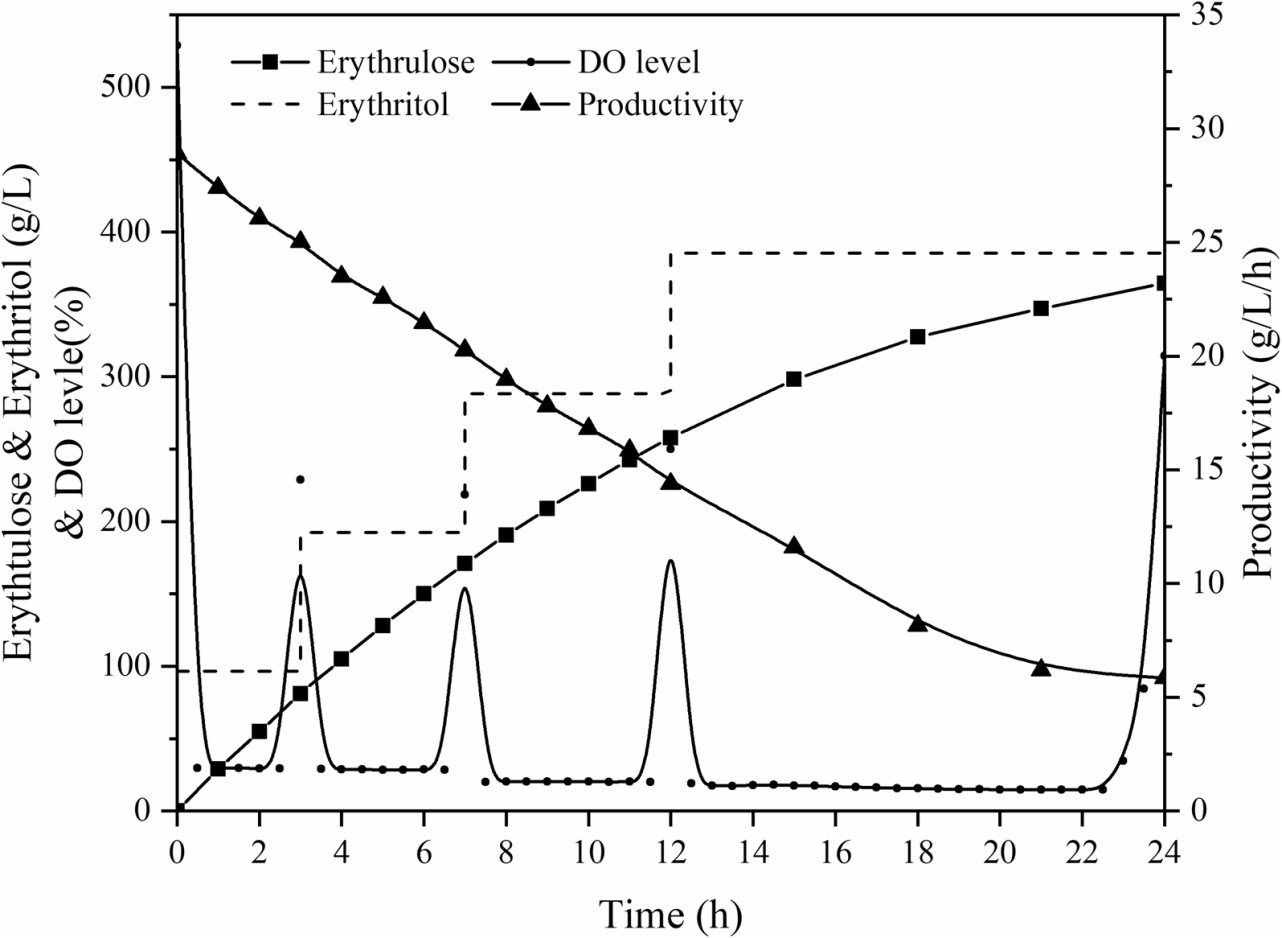
The whole-cell catalysis profile of erythritol in SOS-BR

The bioconversion of erythritol involves mainly the membrane-bound glycerol dehydrogenase of *G. oxydans*, and its catalytic end-product is erythrulose. Erythrulose, a natural 4-C ketose whose aldose form is erythrose, is often used in the cosmetics industry, as a substitute anti-allergic to 1,3-dihydroxyacetone because its structure is similar to dihydroxyacetone. As is shown in Fig. 3, the production and productivity of erythrulose reached 354.7 g/L and 14.8 g/L/h after 24 h of whole-cell fed-batch catalysis. The catalytic results were 46.6% higher than that of Christian Burger et al. which was the highest level. Interestingly, in SOS-BR, the end titer of erythrulose exceeded the inhibitory concentration (about 250 g/L).

Through the regulation of SOS-BR, independent of the kind of membrane-bound dehydrogenase of *G. oxydans* used, the ultrahigh-titer end products could be obtained and the efficient utilization of oxygen could be guaranteed at the same time. Additionally, the oxygen-enriched environment of SOS-BR could break through the conventional inhibitory concentration of some products. Thus, both 1,3-PG and erythritol illustrate the utility and universality of SOS-BR.

### Comparison of physical parameters and bioreaction kinetics between typical bioconversion technologies

**Fig. 4:**
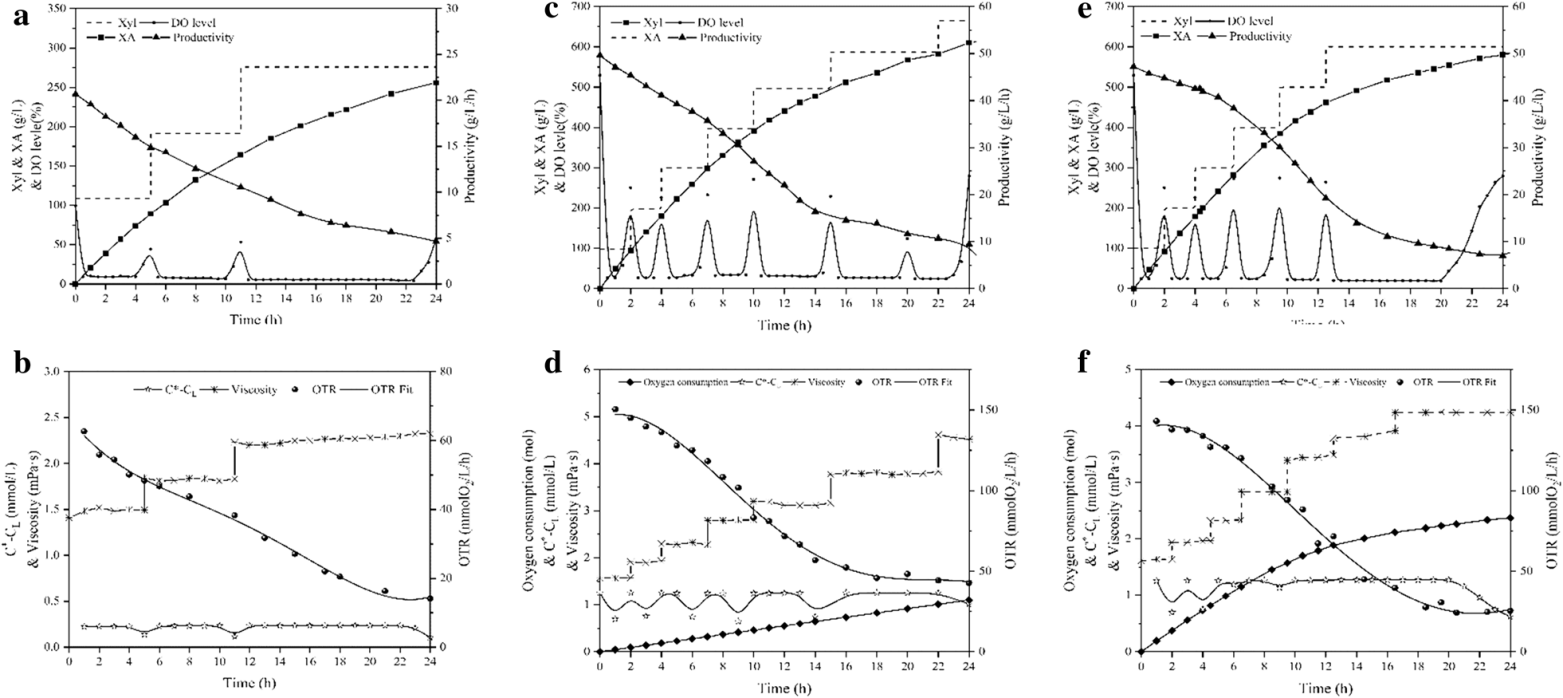
Catalysis profiles of whole-cell catalysis within 72 h, respectively, in AS-BR (**a**, **b**), OS-BR (**c**, **d**) and SOS-BR (**e**, **f**)

Xylonic acid (XA) is mainly derived from a one-step oxidation reaction of xylose and XA has been identified as one of 30 most promising compounds in biomass refining by National Renewable Energy Laboratory (NREL) and Pacific Northwest National Laboratory (PNL) under the U.S. Department of Energy. Precisely, *G. oxydans* is an excellent microbe for the bioproduction of XA from xylose [39]. Moreover, XA is a typical high value-added product of *G. oxydans*, and its catalytic process involves the cells’ membrane-bound aldose dehydrogenase, which combined with 1,3-PG and erythritol, includes all the major enzymes of *G. oxydans*. Hence, the whole-cell catalysis of xylose in bioreactors with different aeration models was carried out and the entire bioprocess was reviewed and compared in detail. Biotransformation profiles of xylose in AS-BR, OS-BR, and SOS-BR are shown in Fig. 4.

As can be seen from Fig. 4a, a total production of 255.8 g XA and the volume productivity of 10.7 g/L/h were achieved by 24-h whole-cell catalysis in AS-BR. However, in the whole bioprocess, expect for the rebound of DO, when the substrate was depleted, the DO level could only be limited to less 10%, which was far below the critical DO level of *G. oxydans* and could not guarantee its normal physiological and catalytic functions. As shown in Fig. 4b, the oxygen driving force was only 0.22–0.23 mmol O_2_/L under ventilation, and the initial maximum OTR was 62.7 mmol O_2_/L/h. When the viscosity of the broth in AS-BR increased to 2.2 mPa s, the DO level dropped to 5%, that severely inhibited the overall metabolism of cells. Visibly, the normal ventilation condition was far from propping up the regular demand of oxygen for high-density whole-cell catalysis. Afterwards, we performed 24-h continuous catalysis in OS-BR under oxygen-enriched atmosphere for the sake of remedying the restrictive bottleneck. As shown in Fig. 4c, within 24 h, the production and productivity of XA increased significantly to 610.2 g/L and 25.4 g/L/h and DO level also increased to 24–26% (20–30% of high-efficiency production level). Moreover, the oxygen driving force heightened to 1.23–1.25 mmol O_2_/L and OTR also increased to 143.1 mmol O_2_/L/h. However, the oxygen consumption increased linearly throughout the bioprocess, with a total consumption of 32.2 mol and an oxygen utilization rate of only 5.3%. Moreover, because the titer of the end-product was more than 600 g/L, the viscosity was high at 4.5 mPa s, increasing the severity of the foam issue. Faced with microbial ‘SOS’, we repeated the whole-cell catalysis of XA with SOS-BR. In a 24-h profile, the accumulation and productivity of XA was 580.1 g/L and 24.2 g/L/h. The other properties, including viscosity, oxygen driving force, and OTR, were similar to OS-BR. Only 1.74 mol O_2_ in the aggregate was consumed within 24 h and the oxygen utilization rate of oxygen was up to 92.6%.

As shown in Table 1, AS-BR was inferior to OS-BR and SOS-BR, which highlighted the importance of oxygen for aerobic microorganisms. For the two bioreactor models in oxygen-enriched environment, although the production and productivity of SOS-BR was slightly lower than OS-BR by 3.6%, the oxygen consumption was saved by 18.5 times and the utilization rate increased by 16.5 times. Furthermore, in SOS-BR, with the increase in oxygen partial pressure, the foam could be squeezed and crushed in the broth, and released oxygen, which improved the utilization rate of oxygen, and fundamentally solved the foam issue. In conclusion, SOS-BR is an excellent bioreactor to overcome the shortcomings of aerobic microorganisms with high-quality utilization of oxygen.

**Table 1:** The performance of XA produced by *G. oxydans*, respectively, in AS-BR, OS-BR and SOS-BR

	Production (g/L)	Productivity (g/L/h)	DO (%)	OTR mmol O_2_/L/h	C* − *C*_L_ mmol O_2_/L	Oxygen consumption (mol)	Utilization (%)
AS-BR	255.8	10.7	< 10	62.2	0.22–0.23	–	–
OS-BR	610.2	25.4	20–30	150.4	1.23–1.25	32.2	5.3
SOS-BR	588.4	24.5	20– 30	143.1	1.26–1.27	1.7	92.6

### Applicability and superiority of SOS-BR for liquid–solid mass transfer

**Fig. 5:**
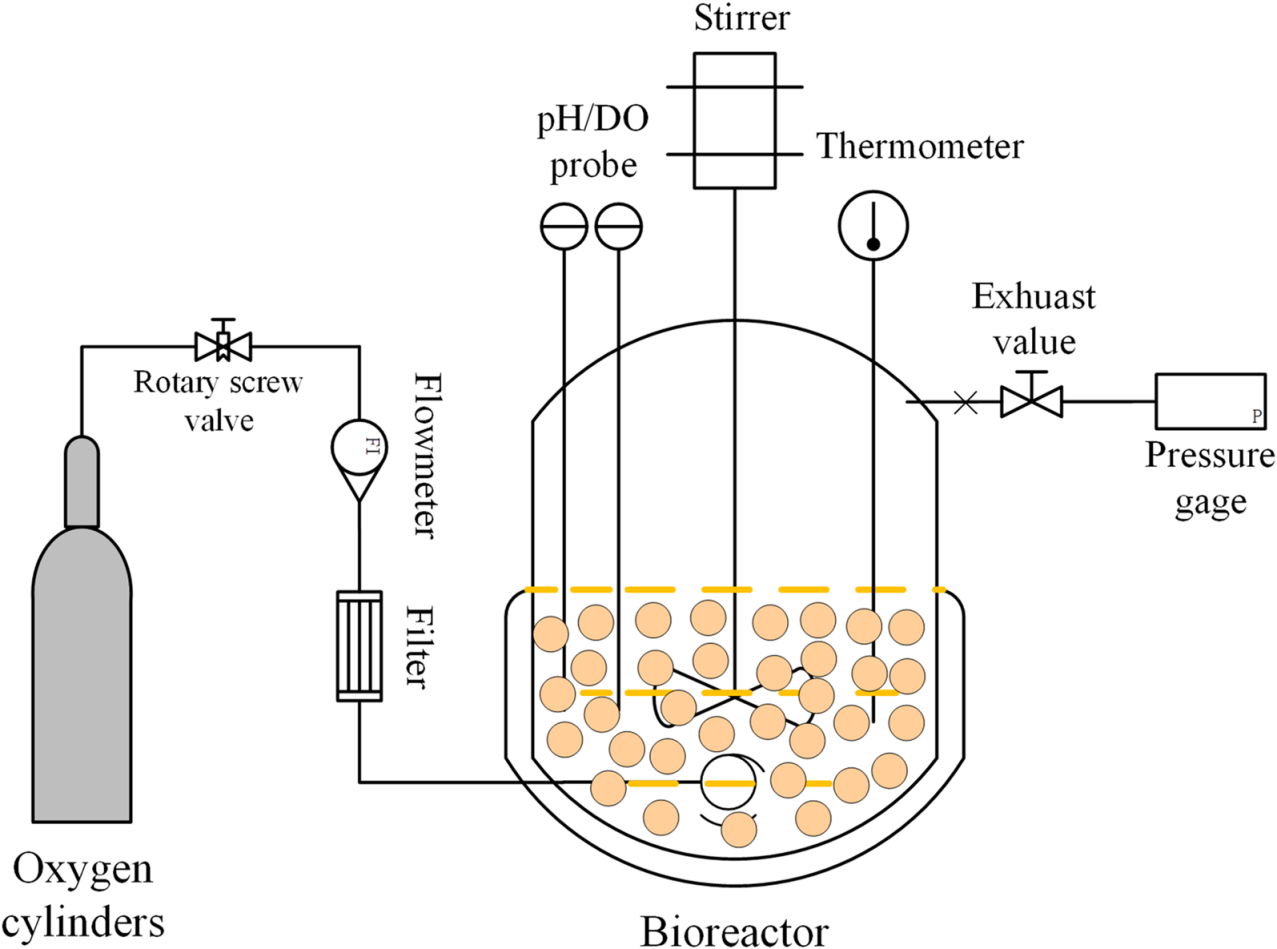
The bioreactor operation models of SOS-BR with immobilized *G. oxydans*

The above experiments on SOS-BR were based on the mass transfer between gas and liquid phase. However, in fact, most of biological products used in industrial production are prepared by cell immobilization technology. Hence, to fully explain the excellence of SOS-BR, it was necessary to study the liquid–solid transfer along with the gas–liquid mass transfer. Figure 5 shows that the whole-cell catalysis was conducted in the bioreactor operation models of SOS-BR with immobilized *G. oxydans* using sodium alginate.

**Fig. 6:**
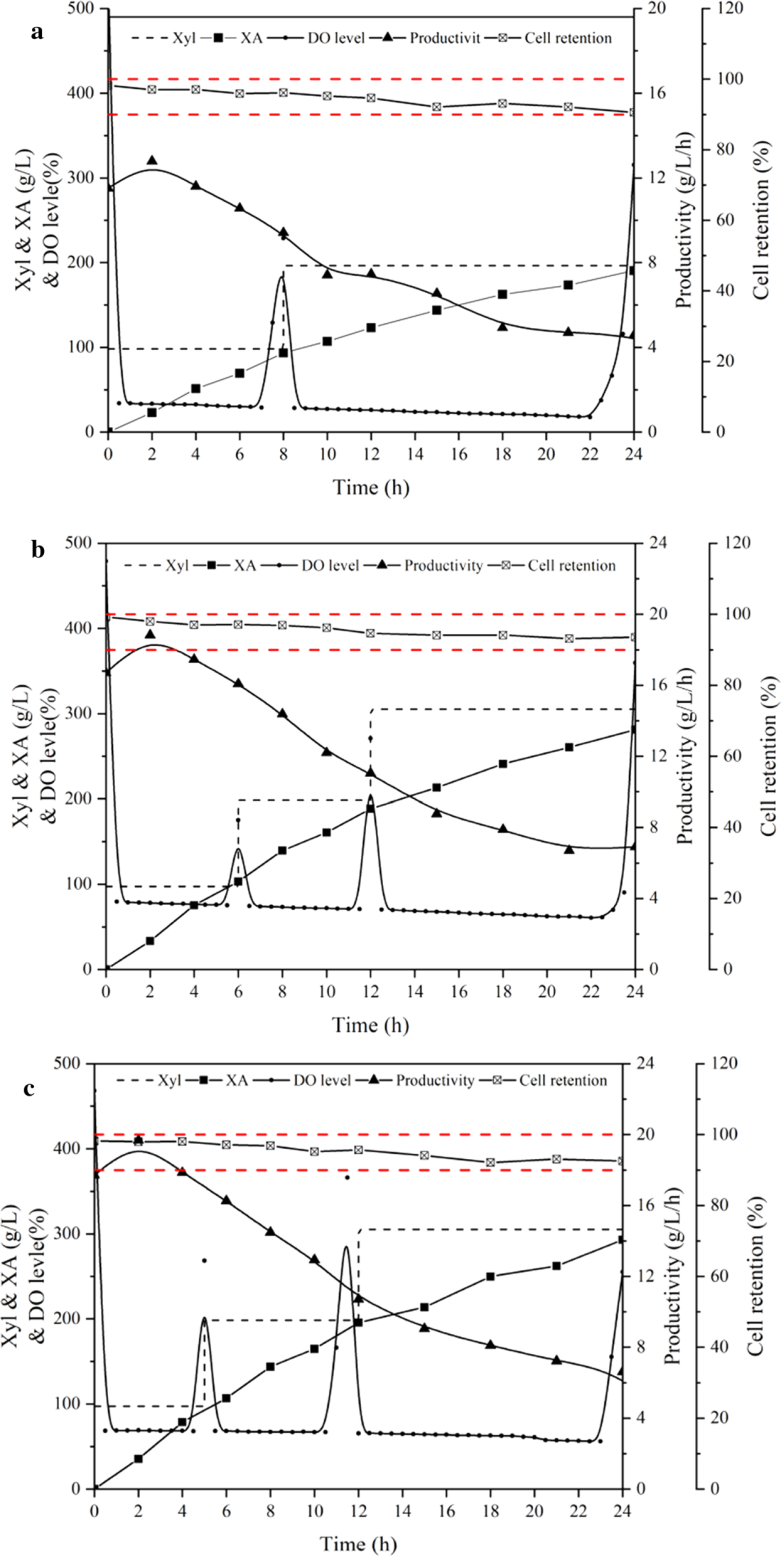
The whole-cell catalysis of immobilized *G. oxydans* in AS-BR, OS-BR, SOS-BR

As is shown in Fig. 6, the comparison of whole-cell catalysis reactions performed in AS-BR, OS-BR, and SOS-BR were investigated. Within 24 h of continuous catalysis, 190.6 g/L, 281.2 g/L, and 292.1 g/L XA were accumulated in three bioreactors with corresponding productivity of 7.9 g/L/h, 11.7 g/L/h, and 12.2 g/L/h, respectively. Noticeably, after the combined immobilization technology, the product concentration of the compressed oxygen-supplied operation model was no longer lower than that of the direct pure-oxygen model. It is speculated that the increase of oxygen partial pressure in SOS-BR made it easier for the nutrient factors such as substrates, nitrogen sources, and oxygen to be compressed to shuttle the immobilized carrier and contact cells to achieve efficient catalysis. The DO levels of free cells and immobilized cells in AS-BR during whole-cell catalysis were monitored and compared. It was found that under the same inoculation and ventilation conditions, the DO level of the immobilized cells was much higher than that of free cells. This did not imply that the DO level of immobilized cells had reached efficient catalytic values, but the immobilized carrier restricted the use of DO in the broth by *G. oxydans,* increasing DO in the broth, which highlighted the importance and necessity of compressed pressure technology in SOS-BR. In addition to investigating the effect of pressurization on mass transfer, it was necessary to evaluate whether SOS-BR could damage the immobilized carrier. Therefore, we selected the most conventional immobilization mode, sodium alginate immobilization technology, to consider the loss of cells. Under normal air or oxygen stirring conditions, the cell retention rate of 24-h whole-cell catalysis was about 93.5%, while the cell retention rate of SOS-BR was insignificantly different, about 92.6%, indicating that the strengthening of oxygen partial pressure had no influence on the conventional immobilized carrier. The outcome showed that SOS-BR could achieve significant mass transfer effect in gas–liquid–solid phases, which could effectively enhance the production capacity while ensuring high-quality utilization of oxygen and inhibition of foam.

## Conclusions

In the field of chemical engineering, this sealed-oxygen supply bioconversion strategy seems to be a kind of SOS slogan to promote biotech and bioengineering developments and industrial applications. The design of SOS-BR could not only enhance the oxygen driving force and OTR, but also solve the foaming issue, thus overcoming the bottleneck of microorganisms in industrial production. Using this biotechnology, high-efficiency of catalysis and high-quality utilization of oxygen has been achieved using *G. oxydans* as catalyst. Finally, the biotechnology of modified bioreactor demonstrates an efficient, pragmatic, and stable biotechnology model, which provides a cost-effective approach for industrialized bio-based fuels and chemical production of aerobic microorganisms.

## Materials and methods

### Microorganism

*Gluconobacter oxydans* NL71 strain (derived from ATCC621) was stored on sorbitol-agar culture medium containing 50 g/L sorbitol, 15 g/L agar, and 5 g/L yeast extract at 4 °C. *G. oxydans* was germinated in a 250-mL Erlenmeyer flask with three baffles containing 50 mL nutrient medium (100 g/L sorbitol and 10 g/L yeast extract) and cultivated in a constant temperature shaker at 30 °C and 220 rpm for 24–36 h. Bacteria were harvested using refrigerated centrifugation (Avanti J-26 XP, Beckman Coulter) at 8000 rpm for 5–10 min as a reserve [6].

### Whole-cell catalysis

The whole-cell catalysis bioprocesses were conducted in a 3.0-L bioreactor (New Brunswick BioFlo 115) including 1 L of culture medium composed of 5 g/L yeast extract, 0.5 g/L MgSO_4_, 1 g/L KH_2_PO_4_, 2 g/L K_2_HPO_4_, 5 g/L (NH_4_)_2_SO_4_ and appropriate amount of substrate. The process was performed through fed-batch operation to avoid substrate inhibition negative-feedback effect. The catalysis broth was stirred at 500 rpm for 24 h and pH was adjusted to 5.5–6.5 using 10% NaOH [40].

Bioprocesses for the biochemical product were performed in three types of bioreactors: air supplied bioreactor (AS-BR), oxygen supplied bioreactor (OS-BR), sealed oxygen supplied bioreactor (SOS-BR). AS-BR was aerated by air compressor, and the airflow rate was regulated at 3 vvm. For OS-BR, pure oxygen (Purity ≥ 99.9%) was fed into the bioreactor with an oxygen cylinder replacing the air compressor, and the ventilation rate was controlled at 0.5 vvm. SOS-BR was connected to an exhaust valve similar to OS-BR. The pressure of the tank was adjusted at 0.03–0.05 MPa. The pressure and the oxygen consumption rate of microorganisms in the bioreactor directly determined the oxygen inflow rate. Foaming was monitored by on-line addition of polyethylene as an antifoam agent in AS-BR and OS-BR, but not in SOS-BR [41].

### Immobilization operation of *G. oxydans*

The immobilized carrier was prepared using 3% sodium alginate dissolved in super-clean water assisted by magnetic stirring at 70 °C for 1–2 h. Then, the fully dissolved sodium alginate colloid was sterilized in autoclave at 121 °C for 15 min and stored at 4 °C [42]. Prepared biomass of 20 OD_600_ *G. oxydans* was aseptically washed into sodium alginate colloid using sterile water. Mixed colloid containing *G. oxydans* was transported by a peristaltic pump to drop the beads into the cross-linking agent, containing 3.5% calcium chloride, automatically with a needle barrel with a diameter of 2–3 mm. Afterwards, the cross-linking agent containing immobilized beads were preserved at 4 °C for 1–2 h. Finally, the cross-linked immobilized beads were washed with deionized water to remove residual calcium chloride and unfixed bacteria on the surface of the carrier and stored in distilled water at 4 °C for further use.

### Determination of parameters in whole-cell catalytic process

Oxygen consumption (mol) was calculated by the pressure in the oxygen cylinder. The formula is as follows:

1 $$V\, = \,\frac{{\left( {G_{2} - G_{1} } \right) \times V_{0} \times g}}{1000},$$

2 $$n = \frac{V}{22.4},$$

where *V* (L) represents the actual gas volume consumption in oxygen cylinders; *V*_0_ (L) represents the total volume in oxygen cylinder (40 L); *G*_1_ and *G*_2_ (MPa) represent real-time pressure in oxygen cylinder before and after whole-cell catalysis, and *g* (m/s^2^) represents gravity acceleration (9.8 m/s^2^).

OTR (mmol O_2_/h/L) is oxygen transfer rate between gas–liquid and liquid–solid phase in the broth. The formula is as follows:

3 $${\text{OTR}}\, = \,k_{\text{L}} \alpha \times \left( {C^{*} - C_{\text{L}} } \right),$$

where C^*^ (mmol/L) is the saturated concentration of dissolved oxygen in broth (mmol/L) and C_L_ (mmol/L) is actual dissolved oxygen concentration in broth; C^*^ − *C*_L_ represents the driving force of oxygen concentration difference, and *K*_L_α (h^−1^) represents oxygen transfer coefficient.

OUR (mmol O_2_/h/L) is oxygen uptake rate of cell reaction system. The formula is as follows:

4 $${\text{OUR}}\,{ = }\,q_{{{\text{O}}_{ 2} }} \times C_{\text{X}} ,$$

where $q_{{{\text{O}}_{ 2} }}$ (mmol/(g h)) represents specific oxygen consumption rate and *C*_x_ (g/L) represents the biomass of *G. oxydans*.

Viscosity (mPa s) was measured using a rheometer (Thermo Scientific HAAKE RheoStress6000).

### Analytical methods

In whole-cell catalysis bioprocess, xylose, erythritol, 1,3-propylene glycol (1,3-PG), and 3-hydroxypropionic acid (3-HPA) were obtained from Aladdin. Xylonic acid (XA) was purchased from TRC-Canada, erythrulose and yeast extract were procured from Sigma. All other chemicals including nutrient salts and sodium alginate were of analytical grade and were commercially available.

The concentration of xylose and XA were detected by high-performance anion-exchange chromatography (HPAEC) coupled with pulsed amperometric detector (Thermo ICS-5000). NaOH (100 mM) was used as mobile phase at flow rate of 0.3 mL/min. The separation column used was CarboPac™ PA200. The titer of erythritol, erythrulose, 1,3-PG and 3-HPA were measured by high-performance liquid chromatography (HPLC) (Agilent 1100 series) equipped with Carbohydrate Ca^++^ 8um HyperRez XP Column and deionized water, after ultrasound, was used as mobile phase at 0.6 mL/min.

Five parallel assays were performed for each experiment.

## Data Availability

All data generated and analyzed in this study are included in this published article.

## Abbreviations

SOS: sealed-oxygen supply
OTR: oxygen transferring rate
XA: xylonic acid
3-HPA: 3-hydroxypropionic acid
NADPH: nicotinamide adenine dinucleotide phosphate
FADH: flavin adenine dinucleotide
ATP: adenosine triphosphate
DO: dissolved oxygen
OUR: oxygen uptake rate
AS-BR: air supplied bioreactor
OS-BR: oxygen supplied bioreactor
SOS-BR: sealed oxygen supplied bioreactor
1,3-PG: 1,3-propylene glycol
HPLC: high performance liquid chromatography
HPAEC: high performance anion-exchange chromatography
OD: optical density
